# Quality of teamwork in multidisciplinary cancer team meetings: A feasibility study

**DOI:** 10.1371/journal.pone.0212556

**Published:** 2019-02-15

**Authors:** David Benjamin Lumenta, Gerald Sendlhofer, Gudrun Pregartner, Marlies Hart, Peter Tiefenbacher, Lars Peter Kamolz, Gernot Brunner

**Affiliations:** 1 Research Unit for Safety in Health, c/o Division of Plastic, Aesthetic and Reconstructive Surgery, Department of Surgery, Medical University of Graz, Graz, Austria; 2 Comprehensive Cancer Center Graz, Medical University of Graz and University Hospital Graz, Graz, Austria; 3 Executive Department for Quality and Risk Management, University Hospital Graz, Graz, Austria; 4 Institute for Medical Informatics, Statistics and Documentation, Medical University of Graz, Graz, Austria; Cambridge University Hospitals NHS trust, UNITED KINGDOM

## Abstract

**Background:**

Tumor boards (TB) play an important role to formulate a management plan for the treatment of patients with a malignancy. Recent evidence suggests that optimally functioning teams (teamwork, communication and decision making) are major prerequisites to conduct efficient TB meetings. The aims of this study were i) to use a readily published tool as a template for the development of a teamwork perspective extended assessment tool and ii) to evaluate the tool in a feasibility study by clinical and non-clinical observers.

**Methods:**

A systematic literature search in four databases revealed the “Metric for the Observation of Decision-making (MODe)” to be consistently used. MODe served as a template for the clinical evaluation, additional, notably teamwork items were integrated, and the resulting tool was tested in a feasibility study in TBs by clinical and non-clinical observers. The percentage of agreement between observers was assessed in a two-step approach: first, agreement of raters on discussion of items by TB members, and second, agreement of raters based on ordinal scale.

**Results:**

In total, 244 patients were discussed in 27 TB sessions, thereof 136 (56%) fast track cases and 108 (44%) complex cases. In 228 (93%) of all cases an agreement for recommendation of a treatment plan was reached. Observers showed in general high agreement on discussion of the items. For the majority of items, the percentage of agreement between the different pairs of rater was similar and mostly high.

**Conclusion:**

A newly developed TB team performance tool using MODe as a template was piloted in a German-speaking country and enabled the assessment of specialized multidisciplinary teams with a special focus on teamwork patterns. The developed assessment tool requires evaluation in a larger collective for validation, and additional assessment whether it can be applied equally by non-clinicians and clinicians.

## Introduction

Multidisciplinary teams (MDT) in tumor boards (TBs) play an increasingly important role for formulating a management plan for patients’ treatments [1,2]. Across the world TBs are implemented, however, required participants differ among countries. For example, in the UK clinical nursing staff specialists take part [3,4]. In Austria, the core TB-team consists of representatives of radiation-oncologists, medical oncologists, radiologists, pathologists, and the involved surgical discipline(s) [5]. However, TBs are open to non-mandatory members like medical students, psychologists, or clinical nursing staff specialists. More advanced TB approaches established the participation of patients with shared decision making, especially when complex treatment options with varying short- or long-term side effects exist [6–8]. In general, the quality of decision making in MDT depends on the quality of information presented, the quality of team performance, the infrastructure for meetings including its organization and logistics [9,10].

In our researched setting, TB-members meet at least bi-monthly in a dedicated pre-reserved location with adequate technological up-to-date equipment (online availability of all clinical parameters and imaging) to discuss clinical cancer cases requiring the interdisciplinary discussion for a management plan. The recommendation is explained to the patient after the meeting, and in case of obtained informed consent realized. The entire processes for TB meetings are standardized, and published in the hospital intranet following standard operating procedures (SOP).

The quality of teamwork, communication and decision making are major prerequisites to conduct efficient TB meetings [11]. Decision-making of teams is influenced by three major factors: i) attendance of core TB members, ii) teamwork, and iii) leadership of the chair [11–15]. In TBs, members have complementary abilities, and the team must ensure that each member’s expertise is appropriately matched to the task, resulting in improved overall team performance [16]. Therefore, it is crucial to improve the understanding of determinants of team performance [16,17].

Before conducting this feasibility study, team performance was not assessed in any TB at our institution, only presence of core TB team members in each TB was thus far the main evaluation parameter for its assessment. The results of attendance of TB-members were forwarded to the chair of the TB and other disciplines in order to improve attendance rates by raising awareness. In sum, more refined evaluations including quality of teamwork, communication, availability of data and decision making were not evaluated previously, but identified as positively benefiting factors for MDTs [3].

The “Metric for the Observation of Decision-making”(MODe) and an adapted version in Germany were considered the most readily-published evidence-based observational tools for TBs, but lacked key aspects of team performance (teamwork, involvement of members, mutual respect, level of agreement, and confounding factors) [18,19]. The aims of this study were i) to use a readily-published tool as a template for the development of a teamwork perspective extended assessment tool and ii) to evaluate the tool in a feasibility study by clinical and non-clinical observers.

## Materials and methods

The study was approved by the ethical board of the Medical University of Graz (vote#: 28–359 ex15/16).

### TB team performance assessment tool

A literature search in four databases (CINAHL, Pubmed, OVID, Embase) was performed to identify a tool for assessing MDT/TB team performance. The following terms were used in the search: “tumor boards OR multidisciplinary”, “cancer meetings OR multidisciplinary”, and “team decision-making OR multidisciplinary team working OR multidisciplinary cancer team OR cancer conference OR case conference AND cancer OR malignancy OR tumor OR carcinoma OR ulcer AND eval OR vali OR measure instrument”. All studies using an assessment tool for MDT/TBs were included. In total, 12 studies using a team performance assessment tool were identified [12,18, 19–26, 27,28].

MODe was used most frequently for evaluation of teamwork and MDT/TB performance [24]. The tool is based on a 5-point Likert-type scale ranging from “high performance” (coded as 5) to “poor performance” (coded as 1). Key aspects of team performance are (i) information presentation to the team covering all relevant domains, (ii) team leadership focusing on aspects of effective and ineffective leadership, and (iii) decision-making and a team decision-making process including level of involvement of different healthcare professional groups [24]. Hahlweg et al. adapted MODe to six variables, namely 1) quality of case history, 2) quality of radiological information, 3) quality of information on comorbidities, 4) whether the case was presented, whether the case was palliative, 5) quality of psychological information, and 6) quality on the information on the patient’s view and preferences. Furthermore the quality of team processes on the case level, quality of team behavior, and medical and treatment uncertainty during the case discussion were assessed [18].

MODe, which contains three categories (“Information” with 6 items, “Discussion” with 8 items, “Outcome” with 1 item) and one additional text item, was used as a template for the development of a wider assessment tool (Table 1). The resulting tool accounted for linguistic and cultural differences, and included key aspects of team performance (teamwork, involvement of team members, mutual respect among team members and level of agreement, and confounding factors). Owing to the simplicity of the terms/items in the German and English language, no further back-translation was provided. Therefore, the study can only be seen as preliminary evidence to support the value of the tool. In particular, our tool differs from MODe in the following aspects:

1. Whereas MODe had separate items for “case history” and “quality of information on co-morbidities”, the new tool merged these two items into “case history and patient data” and comprised name, birth date, diagnosis, co-morbidities, history of presenting complaint, and past medical history, which is in German-speaking countries commonly called “Anamnese”.
2. In addition to the available MODe items, “radiological images (X-ray)” and “histopathological information”, “laboratory results” were added to the tool as within our TBs laboratory results are often a pre-requisite for a TB-registration and are relevant for discussions within a TB.
3. To assess the key aspects of team behavior in detail, “teamwork”, “involvement of team members”, “mutual respect among team members”, “level of agreement among team members”, and “confounding factors” were added to the assessment tool.
4. Cases can be discussed briefly, or, as often occurring, complex cases require detailed discussion among TB-members. Therefore, we distinguished between fast track and complex cases. On the one hand, fast track cases did not require a thorough discussion or case workup by the MDT/TB because the decision could be easily made based on available guidelines (“straightforward decision-making” = “fast track case”). Complex cases, on the other hand, needed elaborate discussion among team members as a result of diverging literature findings (eg. classification schemes, categorization, or treatment recommendations), and such cases were also considered for enrolment in ongoing clinical trials at our institution.

**Table 1:** MTD/TB team performance assessment tool.

**Information**		
**Case history & patient data (item 1)**	5	Comprehensive case history is available
	3	Comprehensive case history is partially available
	1	Comprehensive case history is not available
	0	Comprehensive case history available but not mentioned
**Laboratory results (item 2)**	5	Relevant laboratory results are available
	3	Relevant laboratory results are partially available
	1	Relevant laboratory results are not available
	0	Relevant laboratory results available but not mentioned
**Pathology (item 3)**	5	Relevant histopathological information from pathologist are available
	3	Relevant histopathological information from pathologist are partially available
	1	Relevant histopathological information from pathologist are not available
	0	Relevant histopathological information from pathologist available but not mentioned
**X-ray (item 4)**	5	Relevant radiological images are available
	3	Relevant radiological images are partially available
	1	Relevant radiological images are not available
	0	Relevant radiological images available but not mentioned
**Performance Quality**		
**Contribution moderator (item 5)**	5	Good leadership, is moderating the discussion and supports decision finding with the team
	3	Leadership is not supportive for discussion and decision making is vague
	1	Inadequate leadership, poor moderation and poor decision finding
	0	Not available
**Contribution radiation-oncologist (item 6)**	5	Comprehensive input or input not necessary
	3	Minor input or vague
	1	No input
	0	Not available
**Contribution radiologist (item 7)**	5	Comprehensive input or input not necessary
	3	Minor input or vague
	1	No input
	0	Not available
**Contribution surgeon (item 8)**	5	Comprehensive input or input not necessary
	3	Minor input or vague
	1	No input
	0	Not available
**Contribution pathologist (item 9)**	5	Comprehensive input or input not necessary
	3	Minor input or vague
	1	No input
	0	Not available
**Contribution medical oncologist (item 10)**	5	Comprehensive input or input not necessary
	3	Minor input or vague
	1	No input
	0	Not available
**Communication**		
**Teamwork (item 11)**	5	The team has a cooperative communication style on an expert level
	3	The team has no cooperative communication style on an expert level
	1	One discipline dominates the discussion
	0	Not leviable
**Involvement of team members (item 12)**	5	All available disciplines are actively taking part in the discussion
	3	Not all available disciplines are actively taking part in the discussion
	1	One discipline dominates the discussion
	0	Not leviable
**Mutual Respect among team members (item 13)**	5	Alertness and respectfulness for the one who is speaking
	3	Partial alertness and respectfulness for the one who is speaking
	1	No alertness and respectfulness for the one who is speaking
	0	Not leviable
**Decision making**		
**Level of agreement among team members (item 14)**	5	Therapy plan is concordant within all disciplines
	3	Therapy plan is postponed according to missing data
	1	No or unclear decision making
	0	Not leviable
**Integration of patients’ regards in decision (item 15)**	5	Patients’ regards were integrated in decision making
	1	Patients’ regards were not integrated in decision making
	0	Not leviable
**Confounding factors**		
**Confounding factors (item 16)**	5	Confounding factors were prevented (e.g. phone-calls)
	3	Confounding factors were partially prevented
	1	Confounding factors dominated
	0	Not leviable
Pat ID:		
Day of observation:		
Name of observer:		
Name of TB:		
Fast track case:		
Complex case:		
Duration of the TB (min):		Duration for each case (min):

Finally, the tool consisted of 5 categories (information: items 1–4; performance quality: items 5–10; communication: items 11–13; decision making: items 14–15; others: item 16). We also used the same Likert-type scaling approach as MODe, but added an additional rating category to log whether items were “not available/not mentioned/not assessable (0), thus changing it to a nominal scale. For further information on the tool, see Table 1 and S1 Fig. The usability of the TB team performance assessment tool was then assessed in a feasibility study.

### Feasibility study

Within the Comprehensive Cancer Center Graz (CCC Graz) of the University Hospital Graz, eleven TBs have been installed. Most of the TBs run on a weekly basis with a total of over 4,200 cases per year. Each TB is registered in the electronic patient record system (EPR), and hospital-based information is available in real-time once approved by the chair of a TB. The TB’s attendance and recommendation per case are recorded in the EPR. MDT/TB performance assessments were performed by non-clinical members (a nursing graduate as observer 1, an experienced team member of the management department as observer 2), and a TB meeting physician (summarized as observer 3). The observers were unaware of each other’s ratings during the feasibility study.

The assessment tool was first piloted by observers 1 and 2 for two weeks to test ease of use of the new assessment tool (data not presented). Physicians who were observers in any of the four TBs were instructed in the use of the new tool and tested the assessment tool prior to its use in the feasibility study (data not presented). After the pilot phase, it was the overall goal of the feasibility study to assess approximately 250 patients comparable to the study of Hahlweg et al. [18]. To reach the anticipated amount of patients, four of the eleven available TBs were chosen at random. Each TB chair as well as the TB members were contacted and informed about the nature and raters of the feasibility study with the option to opt-out (= no participation of raters granted). It was anticipated that the feasibility study would last two months to reach the anticipated goal of 250 patients.

### Statistical analysis

The summary of the rating behaviour of the three observers was challenging because of the nominal nature of the data, and we chose to present data in a heat map: showing for each of the 16 items how many percent of respectively observed cases were rated in which rating category (0, 1, 3, or 5) by each of the observers. Additionally, pairwise agreement between the observers was analysed using a two-step approach: First, we assessed how well observers agreed on whether the respective items were discussed/available; this was done by percent agreement on whether an item was rated “0” or not. In a second step, for those cases in which both observers agreed that the respective item was discussed/available, we again assessed their percent agreement and also calculated a weighted version of Cohen’s Kappa coefficient, along with 95% confidence intervals. Due to the highly variable number of cases observed by each of the three observers, we focused on pairwise comparisons rather than a combination of all three. Even though the weighted Kappa coefficient can take into account that ratings in neighbouring categories are closer than a rating of 1 vs. a rating of 5, we decided to present these results only in the supplementary material. This is because the Kappa coefficient considers that observers may agree by chance, and the values are therefore unexpectedly low and difficult to interpret if an item is rated in mainly one category by all raters. All analyses were conducted using R version 3.4.4.

## Results

### Characteristics of observed TBs

In total, 27 TB sessions from four TBs were assessed with the assessment tool (TB1: n = 7; TB2: n = 9; TB 3: n = 5; TB 4: n = 6). In total, 244 patients were discussed (TB1: n = 58; TB 2: n = 59; TB 3: n = 65; TB 4: n = 62), thereof 136 (56%) fast track cases (TB1: n = 29; TB 2: n = 27; TB 3: n = 34; TB 4: n = 46) and 108 (44%) complex cases. In 228 (93%) of all cases an agreement for recommendation of a treatment plan was reached (TB1: 87.9%; TB 2: 93.2%; TB 3: 93.9%; TB 4: 98.4%). In cases where no recommendation was available, further investigations were arranged and the patient was rescheduled for the next TB. The average number of cases within all TBs was 9.6 per session; the average time per TB session was 42 minutes, resulting in an average duration of 4.4 minutes per case. On average, 7.6 TB members were present at each session.

### Observers’ ratings

**Fig 1:**
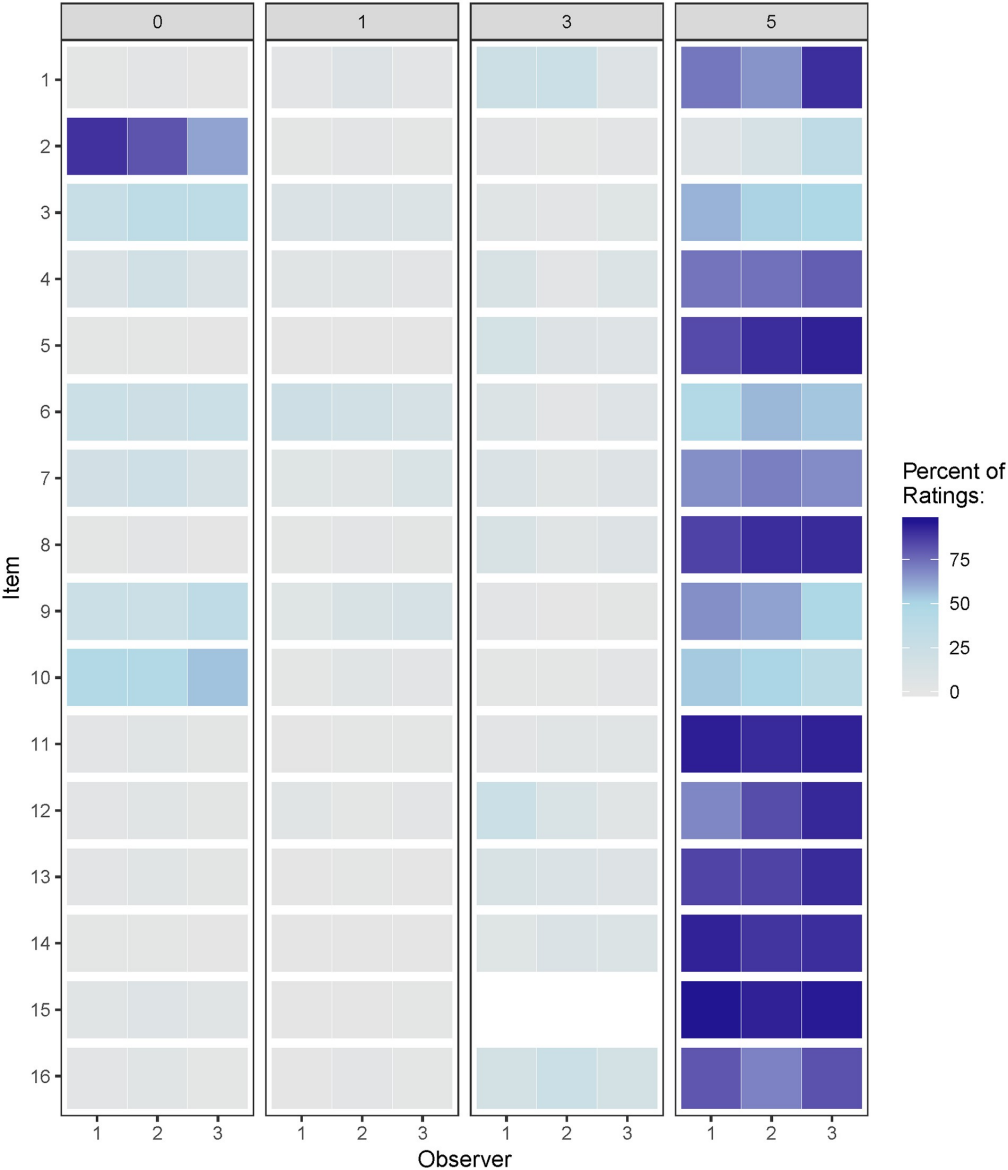
Heat map showing for each of the 16 items how many percent of respectively observed cases were rated “0”, “1”, “3” or “5” by each of the observers.

Whereas observer 1 rated all 244 cases within the feasibility study, observer 2 rated 143 and observer 3 only 126. The heat map (Fig 1) depicting the distribution of each of the observers’ ratings showed that item 2 (laboratory results) was mostly rated as “not discussed” by non-clinical observers, but less often so by the clinician. Many of the remaining items were primarily rated in category 5 (i.e. the best rating) by all observers. However, relevant histopathological information (item 3) as well as the contributions of the radiation-oncologist (item 6), the radiologist (item 7), the pathologist (item 9) and medical oncologist (item 10) was often not mentioned/available. Item 15, which follows a different answer pattern as the other items, assessing whether patients’ regards were considered in the decision-making, was almost exclusively rated as “patients’ regards were integrated”.

### Agreement between observers

According to the first step of the analysis, namely the assessment of how well observers agreed on whether the respective items were discussed (i.e. if they rated them as “0” or not) showed in general high agreements (Table 2). However, for items 2 (laboratory results), 3 (pathology) and 4 (X-ray), non-clinical observers seem to find it harder to assess whether the respective contribution was available. Since only cases in which both respective observers agreed on the item being discussed/available were considered for the analysis in the second step, we present these numbers in Table 3.

**Table 2:** First step of the assessment of observer agreement: Pairwise percent agreement on whether an item was discussed/available (i.e. rated “0” or not).

item	Observers 1 & 2(n = 143)	Observers 1 & 3(n = 126)	Observers 2 & 3(n = 53)
**1**	143 (100.0%)	126 (100.0%)	53 (100.0%)
**2**	132 (92.3%)	93 (73.8%)	40 (75.5%)
**3**	131 (91.6%)	117 (92.9%)	45 (84.9%)
**4**	133 (93.0%)	119 (94.4%)	51 (96.2%)
**5**	143 (100.0%)	126 (100.0%)	53 (100.0%)
**6**	143 (100.0%)	126 (100.0%)	53 (100.0%)
**7**	143 (100.0%)	126 (100.0%)	53 (100.0%)
**8**	143 (100.0%)	126 (100.0%)	53 (100.0%)
**9**	143 (100.0%)	124 (98.4%)	52 (98.1%)
**10**	143 (100.0%)	126 (100.0%)	53 (100.0%)
**11**	143 (100.0%)	126 (100.0%)	53 (100.0%)
**12**	143 (100.0%)	126 (100.0%)	53 (100.0%)
**13**	143 (100.0%)	126 (100.0%)	53 (100.0%)
**14**	143 (100.0%)	126 (100.0%)	53 (100.0%)
**15**	142 (99.3%)	124 (98.4%)	52 (98.1%)
**16**	143 (100.0%)	126 (100.0%)	53 (100.0%)

**Table 3:** Second step of the assessment of observer agreement: Pairwise percent agreement (with 95% confidence interval) on categories 1–5 when there was a consensus that the item was discussed/available.

item	Observers 1 & 2		Observers 1 & 3		Observers 2 & 3	
	Number of observations	Agreement	Number of observations	Agreement	Number of observations	Agreement
**1**	140	88.6 [81.8, 93.1]	126	72.2 [63.4, 79.6]	53	62.3 [47.9, 74.9]
**2**	16	68.8 [41.5, 87.9]	15	86.7 [58.4, 97.7]	11	72.7 [39.3, 92.7]
**3**	90	91.1 [82.7, 95.8]	76	89.5 [79.8, 95.0]	19	94.7 [71.9, 99.7]
**4**	114	84.2 [75.9, 90.1]	111	82.9 [74.3, 89.1]	43	95.3 [82.9, 99.2]
**5**	141	85.1 [77.9, 90.3]	126	89.7 [82.7, 94.2]	53	90.6 [78.6, 96.5]
**6**	111	66.7 [57.0, 75.2]	95	53.7 [43.2, 63.9]	37	59.5 [42.2, 74.8]
**7**	112	81.3 [72.5, 87.8]	108	74.1 [64.6, 81.8]	46	82.6 [68.0, 91.7]
**8**	140	90.7 [84.3, 94.8]	126	82.5 [74.5, 88.5]	53	92.5 [80.9, 97.6]
**9**	107	82.2 [73.4, 88.7]	81	74.1 [62.9, 82.9]	29	82.8 [63.5, 93.5]
**10**	79	88.6 [79.0, 94.3]	57	84.2 [71.6, 92.1]	16	100.0 [75.9, 100.0]
**11**	137	98.5 [94.3, 99.7]	124	99.2 [94.9, 100.0]	51	100.0 [91.3, 100.0]
**12**	137	70.8 [62.3, 78.1]	124	71.8 [62.9, 79.3]	51	90.2 [77.8, 96.3]
**13**	137	92.0 [85.8, 95.7]	124	94.4 [88.3, 97.5]	51	96.1 [85.4, 99.3]
**14**	141	97.9 [93.4, 99.4]	126	96.0 [90.5, 98.5]	53	96.2 [85.9, 99.3]
**15**	134	100.0 [96.5, 100.0]	120	100.0 [96.1, 100.0]	48	100.0 [90.8, 100.0]
**16**	138	76.8 [68.7, 83.4]	125	80.8 [72.6, 87.1]	52	78.8 [64.9, 88.5]

In the second step of the analysis, we assessed how well observers agreed on the ordinal part of the answer categories. Considering that for some items (e.g. items 2, 3, 10) only a small fraction of the initially observed cases were available for this part of the analysis (Table 3), we see that for most items the percent agreement between the different pairs of raters is similar and mostly high. No clear pattern emerged as to which two raters agreed with each other the most. Agreement values for all, complex and fast track cases analysed separately are shown in S1, S2 and S3 Tables.

## Discussion

The readily-published MODe was used as best-practice template for clinical items in our tool. To account for soft skill evaluation we included key aspects of team performance [16]. Items of the original MODe were translated into German and each of these was adapted to account for linguistic and cultural differences in our setting. According to the agreement between observers, differences emerged for a few items, however, it cannot be ruled out that these differences may be due to observers’ background. Overall, the assessment tool demonstrated good agreement especially on soft skill items and ease of use for the observers, but also showed that the newly added laboratory result item was lacking this kind of agreement.

The adapted tool accounted for advances in evaluating teams [29], previously not integrated in the original MODe. For teams, communication can have a great impact on outcome, and this fact was given special attention in the adapted tool [4]. Important determinants for effective TBs are a cooperative and communicative style, commitment of TB members to take part in the discussion, and attentiveness as well as respect [16]. Individual disturbance factors, dominance of certain TB members, or the quality of discussions can have a great influence on the team performance quality, which can be easily assessed with the newly developed assessment tool.

Overall, differences between clinical and non-clinical observers were mostly seen for “presentation of the case history & patient data” and “laboratory results”, whereas all observers were at variance regarding the “contribution of the radiation-oncologist”, and observer 1 and 2 rated “involvement of all team members” differently than the physician. This indicates that for these items a medical background is required for reliable evaluation. For example, not all laboratory values or case history details required mentioning in order to come to a conclusion. This might have been clear to an observer with a medical background but missed by an observer without such knowledge. Therefore, the respective item might have been rated more favourably by the physician. This phenomenon was supposedly more or less prominent depending on the complexity of the cases discussed. For complex cases, the involvement of each relevant MDT/TB member is necessary, whereas for fast track cases the decision-making process is straightforward, and involvement of every MDT/TB member is not necessarily required.

Before this study, evaluation of TBs was only based on analysing EPR data on team member presence in our institution. The fact that non-clinicians showed mostly similar results when using the adapted assessment tool was of special interest for resource planning of quality and risk management departments, often tasked to measure efficiency of processes [16, 17]. Our results suggest that either involvement of clinical or non-clinical observers can lead to the same conclusions for many of the assessed items [21]. Our interpretation of the generally well-prepared MDT/TB meetings suggested predominantly good agreement among observers and the importance of team aspects previously not included in MDT/TB evaluations for overall MDT/TB performance.

In the future, we plan to test the tool in TBs by non-clinical and clinical observers to gain more insights for further adaptation. Our current results already indicated that the newly added item (laboratory results) was not used frequently, and can be omitted. After a final adaptation, for a wide-scale implementation, dedicated physicians of each TB will then be supervised in the use of the assessment tool. It is also planned for a member of the management department to use the tool routinely to collect data and reach out for immediate feedback to each moderator of a TB. It is the vision that the presented assessment tool will become an integral part of TB evaluation and that feedback loops aid improving TB performance.

Limitations of our study include the single institution setting and choice of observers, which did not include all professional groups but subsumed different physicians as “observer 3”. Owing to the simplicity of the terms/items in the German and English language, no further back-translation was provided. Therefore, the study can only be seen as preliminary evidence to support the value of the tool. The assessment tool might be suitable for routine application; however, it requires further investigation in larger samples and different centres. Furthermore, it is questionable, if also non-clinical observers can use the assessment tool for items which are mainly physician driven.

## Conclusions

In conclusion, a readily-published and evidence-based observational tool for assessing MDT was identified, translated into the German language, and adaptations to account for previously ignored aspects like communication style as well as team rules and culture were implemented. According to the results of 244 patients in TBs observed by three independent observers, an overall high level of agreement among observers for the adapted assessment tool, notably for the new items (items 11–14 and 16), was achieved. The developed assessment tool requires evaluation in a larger collective for validation, and additional assessment whether it can be applied equally by non-clinicians and clinicians. It is also of notable interest whether an integrated feedback mechanism to TB-members immediately after conclusion of a TB can have an influence on (future) team performance.

## Supporting information

S1 Fig Tool as used in the feasibility study. (PDF)

S1 Table Rate of agreement and Kappa coefficient for complex cases between observers (obs.). (DOCX)

S2 Table Rate of agreement and Kappa coefficient between observers (obs.) for fast track cases. (DOCX)

S3 Table Rate of agreement and Kappa coefficient between observers (obs.) for all cases. (DOCX)
