# Supplementary material for: Quality of teamwork in multidisciplinary cancer team meetings: A feasibility study
Source: PLoS One. 2019 Feb 15;14(2):e0212556. doi: 10.1371/journal.pone.0212556 (PMC6377131; doi:10.1371/journal.pone.0212556)
Supplement: S1 Fig — (PDF) [file pone.0212556.s001.pdf]

Suppl. File 3: The modified MODE translated as German version

Assessment Instrument für Beobachtung von Tumorboards

Vorraussetzung	1 Co-Morbiditäten und Patienten-daten	5	Erforderlichen Patientendaten, Patientenvorgeschichte und Co-Morbiditäten sind vorhanden	Durchführungsqualität	6 Beitrag Radio-onkologe	5	Präziser und fachlich guter Beitrag der Fachdisziplin/Beitrag nicht notwendig
		3	Erforderliche Daten sind teilweise vorhanden			3	Nötigster Beitrag der Fachdisziplin
		1	Erforderliche Daten sind nicht vorhanden			1	Kein Beitrag der Fachdisziplin
		0	Nicht erwähnt			0	Nicht anwesend
	2 Labor-befunde	5	Aktuelle relevante Laborbefunde sind vorhanden		7 Beitrag Radiologe	5	Präziser und fachlich guter Beitrag der Fachdisziplin/Beitrag nicht notwendig
		3	Laborbefunde sind teilweise vorhanden			3	Nötigster Beitrag der Fachdisziplin
		1	Laborbefunde sind nicht vorhanden			1	Kein Beitrag der Fachdisziplin
		0	Nicht erwähnt			0	Nicht anwesend
	3 Pathologie-befunde	5	Relevante histopathologische Informationen sind von einem Pathologen vorhanden		8 Beitrag APA	5	Präziser und fachlich guter Beitrag der Fachdisziplin/Beitrag nicht notwendig
		3	Relevante Histopathologische Informationen sind teilweise vorhanden			3	Nötigster Beitrag der Fachdisziplin
		1	Relevante histopathologischen Informationen sind nicht vorhanden			1	Kein Beitrag der Fachdisziplin
		0	Nicht erwähnt			0	Nicht anwesend
	4 Röntgen-befunde	5	Relevante radiologische Bilder sind von einem Radiologen vorhanden		9 Beitrag Pathologe	5	Präziser und fachlich guter Beitrag der Fachdisziplin/Beitrag nicht notwendig
		3	Relevante radiologische Informationen sind teilweise vorhanden			3	Nötigster Beitrag der Fachdisziplin
		1	Radiologische Informationen sind nicht vorhanden			1	Kein Beitrag der Fachdisziplin
		0	Nicht erwähnt			0	Nicht anwesend
Durchführungs-qualität	5 Beitrag Moderator	5	Gute Leitung der Team-Diskussion und der Entscheidungsfindung		10 Beitrag Onkologe	5	Präziser und fachlich guter Beitrag der Fachdisziplin/Beitrag nicht notwendig
		3	Die Leitung durch den Moderator ist nicht unterstützend für die Diskussion und Entscheidungsfindung			3	Nötigster Beitrag der Fachdisziplin
		1	Inadäquate Leitung der Diskussion und Entscheidungsfindung			1	Kein Beitrag der Fachdisziplin
		0	Nicht anwesend			0	Nicht anwesend

Erstellt am: 14.03.2016, Version 1

Patientenfallnummer: __________
Datum der Beobachtung:__________

Name des Beobacherts:__________________________
Tumorboard:__________________________

Assessment Instrument für Beobachtung von Tumorboards

Bereich	Item	Punkte	Beschreibung
Kommunikation	11 Teamarbeit	5	Das Team hat einen kooperativen kommunikativen Stil auf fachlicher Ebene
		3	Das Team diskutiert nicht auf fachlicher Ebene
		1	Eine Fachdisziplin dominiert die Diskussion
		0	Nicht erhebbar
	12 Einbezug von anwesenden Fach-disziplinen	5	Alle erforderlichen Fachdisziplinen werden aktiv in die Diskussion involviert bzw. miteinbezogen
		3	Eine Fachdisziplin wird nicht in die Diskussion involviert bzw. miteinbezogen
		1	Eine Fachdisziplin dominiert die Diskussion/Gesprächsführung
		0	Nicht erhebbar
	13 Respekt	5	Es herrscht Aufmerksamkeit und Respekt für die Person, die spricht
		3	Keine Aufmerksamkeit für Beiträge
		1	Beiträge werden nicht respektiert bzw. ignoriert
		0	Nicht erhebbar
Entscheidung	14 Ent-scheidung	5	Es wird einstimmig über die Therapieempfehlung entschieden
		3	Die Entscheidung der Verschiebung auf das nächste Tumorboard aufgrund fehlender Befunde/zu früher Anmeldung
		1	Keine oder unklare Entscheidung
		0	Nicht erhebbar
	15 Patienten-zentrierte Ent-scheidung?	Ja	Patientenwünsche wurden in die Entscheidung integriert
		Nein	Patientenwünsche wurden nicht in die Entscheidung integriert
			Nicht erhebbar
Störfaktoren	16 Stör-faktoren	5	Störungen werden vermieden/treten nicht auf (z.B.: Telefongespräch)
		3	Störungen treten teilweise auf
		1	Störungen treten häufig auf
		0	Nicht erhebbar

Komplexer Fall ☐

Fast track ☐

Erstellt am: 14.03.2016, Version 1

Patientenfallnummer: __________

Datum der Beobachtung:__________

Name des Beobacherts:__________________________

Tumorboard:__________________________
