# Supplementary material for: Quality of teamwork in multidisciplinary cancer team meetings: A feasibility study
Source: PLoS One. 2019 Feb 15;14(2):e0212556. doi: 10.1371/journal.pone.0212556 (PMC6377131; doi:10.1371/journal.pone.0212556)
Supplement: S1 Table — (DOCX) [file pone.0212556.s002.docx]

**S1 Table:** Rate of agreement and Kappa coefficient for complex cases between observers (obs.)

| item | measure | estimate_1vs2 | estimate_1vs3 | estimate_2vs3 |
| --- | --- | --- | --- | --- |
| 1 | Agreement | 87.7 [76.6, 94.2] | 77.0 [64.2, 86.5] | 69.2 [48.1, 84.9] |
|  | Kappa | 0.83 [0.83, 0.83] | 0.45 [-0.24, 1.00] | 0.19 [-1.00, 1.00] |
| 2 | Agreement | 50.0 [23.7, 76.3] | 80.0 [44.2, 96.5] | 62.5 [25.9, 89.8] |
|  | Kappa | 0.07 [0.07, 0.07] | 0.55 [0.55, 0.55] | -0.20 [-1.00, 1.00] |
| 3 | Agreement | 90.0 [75.4, 96.7] | 81.6 [65.1, 91.7] | 100.0 [62.9, 100.0] |
|  | Kappa | 0.83 [0.83, 0.83] | 0.78 [0.78, 0.78] | 1.00 [1.00, 1.00] |
| 4 | Agreement | 80.3 [67.8, 89.0] | 81.0 [68.2, 89.7] | 92.3 [73.4, 98.7] |
|  | Kappa | 0.43 [-0.56, 1.00] | 0.41 [-1.00, 1.00] | 0.00 [-1.00, 1.00] |
| 5 | Agreement | 87.7 [76.6, 94.2] | 86.9 [75.2, 93.8] | 88.5 [68.7, 97.0] |
|  | Kappa | 0.44 [-1.00, 1.00] | 0.37 [-1.00, 1.00] | 0.51 [-1.00, 1.00] |
| 6 | Agreement | 67.3 [52.3, 79.6] | 58.1 [42.2, 72.6] | 68.8 [41.5, 87.9] |
|  | Kappa | 0.25 [-0.17, 0.67] | 0.29 [-0.04, 0.63] | 0.31 [-0.54, 1.00] |
| 7 | Agreement | 80.7 [67.7, 89.5] | 85.2 [72.3, 92.9] | 84.6 [64.3, 95.0] |
|  | Kappa | 0.36 [-0.40, 1.00] | 0.72 [0.72, 0.72] | 0.49 [-0.54, 1.00] |
| 8 | Agreement | 95.4 [86.2, 98.8] | 85.2 [73.3, 92.6] | 84.6 [64.3, 95.0] |
|  | Kappa | 0.75 [0.75, 0.75] | -0.07 [-1.00, 1.00] | -0.08 [-1.00, 1.00] |
| 9 | Agreement | 81.1 [67.6, 90.1] | 76.3 [59.4, 88.0] | 87.5 [60.4, 97.8] |
|  | Kappa | 0.45 [-0.02, 0.91] | 0.36 [-0.25, 0.96] | 0.60 [0.54, 0.66] |
| 10 | Agreement | 100.0 [86.3, 100.0] | 100.0 [82.8, 100.0] | 100.0 [46.3, 100.0] |
|  | Kappa | - | 1.00 [1.00, 1.00] | - |
| 11 | Agreement | 100.0 [93.0, 100.0] | 100.0 [92.6, 100.0] | 100.0 [84.0, 100.0] |
|  | Kappa | 1.00 [1.00, 1.00] | 1.00 [1.00, 1.00] | 1.00 [1.00, 1.00] |
| 12 | Agreement | 72.3 [59.6, 82.3] | 68.9 [55.6, 79.8] | 92.3 [73.4, 98.7] |
|  | Kappa | 0.22 [-0.92, 1.00] | 0.17 [-1.00, 1.00] | 0.63 [0.63, 0.63] |
| 13 | Agreement | 87.7 [76.6, 94.2] | 91.8 [81.2, 96.9] | 92.3 [73.4, 98.7] |
|  | Kappa | 0.58 [0.58, 0.58] | 0.62 [0.62, 0.62] | 0.00 [-1.00, 1.00] |
| 14 | Agreement | 96.9 [88.4, 99.5] | 96.7 [87.6, 99.4] | 96.2 [78.4, 99.8] |
|  | Kappa | 0.73 [0.73, 0.73] | 0.73 [0.73, 0.73] | 0.84 [0.84, 0.84] |
| 15 | Agreement | 100.0 [92.9, 100.0] | 100.0 [92.5, 100.0] | 100.0 [83.4, 100.0] |
|  | Kappa | - | - | - |
| 16 | Agreement | 67.7 [54.8, 78.5] | 75.4 [62.4, 85.2] | 76.9 [55.9, 90.2] |
|  | Kappa | 0.37 [-0.20, 0.94] | 0.38 [-0.51, 1.00] | 0.20 [-1.00, 1.00] |
