# Supplementary material for: Quality of teamwork in multidisciplinary cancer team meetings: A feasibility study
Source: PLoS One. 2019 Feb 15;14(2):e0212556. doi: 10.1371/journal.pone.0212556 (PMC6377131; doi:10.1371/journal.pone.0212556)
Supplement: S2 Table — (DOCX) [file pone.0212556.s003.docx]

**S2 Table:** Rate of agreement and Kappa coefficient for fast track cases between observers (obs.)

| item | measure | estimate_1vs2 | estimate_1vs3 | estimate_2vs3 |
| --- | --- | --- | --- | --- |
| 1 | Agreement | 89.3 [79.5, 95.0] | 67.7 [54.8, 78.5] | 55.6 [35.6, 74.0] |
|  | Kappa | 0.75 [0.75, 0.75] | 0.31 [-0.58, 1.00] | 0.30 [-0.19, 0.79] |
| 2 | Agreement | 100.0 [51.7, 100.0] | 100.0 [46.3, 100.0] | 100.0 [31.0, 100.0] |
|  | Kappa | 1.00 [1.00, 1.00] | 1.00 [1.00, 1.00] | 1.00 [1.00, 1.00] |
| 3 | Agreement | 92.0 [79.9, 97.4] | 97.4 [84.6, 99.9] | 90.0 [54.1, 99.5] |
|  | Kappa | 0.73 [0.73, 0.73] | 0.98 [0.98, 0.98] | 0.74 [0.74, 0.74] |
| 4 | Agreement | 88.7 [76.3, 95.3] | 84.9 [71.9, 92.8] | 100.0 [77.1, 100.0] |
|  | Kappa | 0.04 [-1.00, 1.00] | 0.71 [0.71, 0.71] | - |
| 5 | Agreement | 82.9 [72.2, 90.2] | 92.3 [82.2, 97.1] | 92.6 [74.2, 98.7] |
|  | Kappa | 0.36 [-1.00, 1.00] | 0.51 [-1.00, 1.00] | 0.47 [-1.00, 1.00] |
| 6 | Agreement | 66.1 [52.9, 77.4] | 50.0 [36.9, 63.1] | 52.4 [30.3, 73.6] |
|  | Kappa | 0.33 [0.02, 0.63] | -0.05 [-0.57, 0.47] | -0.04 [-0.96, 0.88] |
| 7 | Agreement | 81.8 [68.6, 90.5] | 63.0 [48.7, 75.4] | 80.0 [55.7, 93.4] |
|  | Kappa | 0.45 [-0.16, 1.00] | 0.01 [-0.93, 0.95] | 0.53 [0.11, 0.95] |
| 8 | Agreement | 86.7 [76.4, 93.1] | 80.0 [67.9, 88.5] | 100.0 [84.5, 100.0] |
|  | Kappa | 0.56 [0.32, 0.81] | 0.57 [0.43, 0.71] | 1.00 [1.00, 1.00] |
| 9 | Agreement | 83.3 [70.2, 91.6] | 72.1 [56.1, 84.2] | 76.9 [46.0, 93.8] |
|  | Kappa | 0.41 [-0.08, 0.90] | 0.17 [-0.68, 1.00] | 0.51 [0.01, 1.00] |
| 10 | Agreement | 81.3 [66.9, 90.6] | 72.7 [54.2, 86.1] | 100.0 [67.9, 100.0] |
|  | Kappa | 0.15 [-1.00, 1.00] | -0.09 [-1.00, 1.00] | 1.00 [1.00, 1.00] |
| 11 | Agreement | 97.2 [89.4, 99.5] | 98.4 [90.3, 99.9] | 100.0 [83.4, 100.0] |
|  | Kappa | 0.88 [0.88, 0.88] | 0.93 [0.93, 0.93] | 1.00 [1.00, 1.00] |
| 12 | Agreement | 69.4 [57.3, 79.5] | 74.6 [61.8, 84.4] | 88.0 [67.7, 96.8] |
|  | Kappa | 0.34 [-0.24, 0.93] | 0.32 [-0.85, 1.00] | 0.54 [-0.14, 1.00] |
| 13 | Agreement | 95.8 [87.5, 98.9] | 96.8 [88.0, 99.4] | 100.0 [83.4, 100.0] |
|  | Kappa | 0.71 [0.71, 0.71] | 0.73 [0.73, 0.73] | 1.00 [1.00, 1.00] |
| 14 | Agreement | 98.7 [91.9, 99.9] | 95.4 [86.2, 98.8] | 96.3 [79.1, 99.8] |
|  | Kappa | 0.94 [0.94, 0.94] | 0.74 [0.74, 0.74] | 0.87 [0.87, 0.87] |
| 15 | Agreement | 100.0 [93.5, 100.0] | 100.0 [92.5, 100.0] | 100.0 [82.2, 100.0] |
|  | Kappa | - | - | - |
| 16 | Agreement | 84.9 [74.2, 91.9] | 85.9 [74.5, 93.0] | 80.8 [60.0, 92.7] |
|  | Kappa | 0.37 [-1.00, 1.00] | 0.24 [-1.00, 1.00] | 0.50 [-0.41, 1.00] |
