# Supplementary material for: Quality of teamwork in multidisciplinary cancer team meetings: A feasibility study
Source: PLoS One. 2019 Feb 15;14(2):e0212556. doi: 10.1371/journal.pone.0212556 (PMC6377131; doi:10.1371/journal.pone.0212556)
Supplement: S3 Table — (DOCX) [file pone.0212556.s004.docx]

**S3 Table:** Rate of agreement and Kappa coefficient for all cases between observers (obs.)

| item | measure | estimate_1vs2 | estimate_1vs3 | estimate_2vs3 |
| --- | --- | --- | --- | --- |
| 1 | Agreement | 88.6 [81.8, 93.1] | 72.2 [63.4, 79.6] | 62.3 [47.9, 74.9] |
|  | Kappa | 0.79 [0.79, 0.79] | 0.37 [-0.21, 0.95] | 0.27 [-0.35, 0.88] |
| 2 | Agreement | 68.8 [41.5, 87.9] | 86.7 [58.4, 97.7] | 72.7 [39.3, 92.7] |
|  | Kappa | 0.45 [-0.14, 1.00] | 0.82 [0.82, 0.82] | 0.66 [0.66, 0.66] |
| 3 | Agreement | 91.1 [82.7, 95.8] | 89.5 [79.8, 95.0] | 94.7 [71.9, 99.7] |
|  | Kappa | 0.76 [0.76, 0.76] | 0.87 [0.87, 0.87] | 0.79 [0.79, 0.79] |
| 4 | Agreement | 84.2 [75.9, 90.1] | 82.9 [74.3, 89.1] | 95.3 [82.9, 99.2] |
|  | Kappa | 0.24 [-1.00, 1.00] | 0.59 [0.59, 0.59] | 0.00 [-1.00, 1.00] |
| 5 | Agreement | 85.1 [77.9, 90.3] | 89.7 [82.7, 94.2] | 90.6 [78.6, 96.5] |
|  | Kappa | 0.39 [-0.72, 1.00] | 0.43 [-1.00, 1.00] | 0.49 [-1.00, 1.00] |
| 6 | Agreement | 66.7 [57.0, 75.2] | 53.7 [43.2, 63.9] | 59.5 [42.2, 74.8] |
|  | Kappa | 0.29 [0.04, 0.54] | 0.09 [-0.25, 0.43] | 0.10 [-0.57, 0.76] |
| 7 | Agreement | 81.3 [72.5, 87.8] | 74.1 [64.6, 81.8] | 82.6 [68.0, 91.7] |
|  | Kappa | 0.40 [-0.11, 0.90] | 0.33 [-0.14, 0.80] | 0.52 [0.12, 0.92] |
| 8 | Agreement | 90.7 [84.3, 94.8] | 82.5 [74.5, 88.5] | 92.5 [80.9, 97.6] |
|  | Kappa | 0.62 [0.62, 0.62] | 0.45 [-0.42, 1.00] | 0.80 [0.80, 0.80] |
| 9 | Agreement | 82.2 [73.4, 88.7] | 74.1 [62.9, 82.9] | 82.8 [63.5, 93.5] |
|  | Kappa | 0.42 [0.07, 0.77] | 0.26 [-0.26, 0.78] | 0.56 [0.30, 0.82] |
| 10 | Agreement | 88.6 [79.0, 94.3] | 84.2 [71.6, 92.1] | 100.0 [75.9, 100.0] |
|  | Kappa | 0.18 [-1.00, 1.00] | 0.22 [-1.00, 1.00] | 1.00 [1.00, 1.00] |
| 11 | Agreement | 98.5 [94.3, 99.7] | 99.2 [94.9, 100.0] | 100.0 [91.3, 100.0] |
|  | Kappa | 0.90 [0.90, 0.90] | 0.94 [0.94, 0.94] | 1.00 [1.00, 1.00] |
| 12 | Agreement | 70.8 [62.3, 78.1] | 71.8 [62.9, 79.3] | 90.2 [77.8, 96.3] |
|  | Kappa | 0.29 [-0.26, 0.85] | 0.24 [-0.64, 1.00] | 0.57 [0.19, 0.94] |
| 13 | Agreement | 92.0 [85.8, 95.7] | 94.4 [88.3, 97.5] | 96.1 [85.4, 99.3] |
|  | Kappa | 0.63 [0.63, 0.63] | 0.67 [0.67, 0.67] | 0.65 [0.65, 0.65] |
| 14 | Agreement | 97.9 [93.4, 99.4] | 96.0 [90.5, 98.5] | 96.2 [85.9, 99.3] |
|  | Kappa | 0.88 [0.88, 0.88] | 0.74 [0.74, 0.74] | 0.85 [0.85, 0.85] |
| 15 | Agreement | 100.0 [96.5, 100.0] | 100.0 [96.1, 100.0] | 100.0 [90.8, 100.0] |
|  | Kappa | - | - | - |
| 16 | Agreement | 76.8 [68.7, 83.4] | 80.8 [72.6, 87.1] | 78.8 [64.9, 88.5] |
|  | Kappa | 0.40 [-0.21, 1.00] | 0.35 [-0.80, 1.00] | 0.37 [-0.99, 1.00] |
